# Chronic High Fructose Intake Reduces Serum 1,25 (OH)_2_D_3_ Levels in Calcium-Sufficient Rodents

**DOI:** 10.1371/journal.pone.0093611

**Published:** 2014-04-09

**Authors:** Veronique Douard, Chirag Patel, Jacklyn Lee, Phuntila Tharabenjasin, Edek Williams, J. Christopher Fritton, Yves Sabbagh, Ronaldo P. Ferraris

**Affiliations:** 1 Department of Pharmacology and Physiology, New Jersey Medical School, Rutgers Biomedical and Health Sciences (RBHS), Newark, New Jersey, United States of America; 2 Department of Biomedical Engineering, Rutgers University, Piscataway, New Jersey, United States of America; 3 Department of Orthopaedics, New Jersey Medical School, RBHS, Newark, New Jersey, United States of America; 4 Tissue Protection and Repair, Sanofi-Genzyme R&D Center, Genzyme, a Sanofi Company, Framingham, Massachusetts, United States of America; Paris Institute of Technology for Life, Food and Environmental Sciences, France

## Abstract

Excessive fructose consumption inhibits adaptive increases in intestinal Ca^2+^ transport in lactating and weanling rats with increased Ca^2+^ requirements by preventing the increase in serum levels of 1,25(OH)_2_D_3_. Here we tested the hypothesis that chronic fructose intake decreases 1,25(OH)_2_D_3_ levels independent of increases in Ca^2+^ requirements. Adult mice fed for five wk a high glucose-low Ca^2+^ diet displayed expected compensatory increases in intestinal and renal Ca^2+^ transporter expression and activity, in renal CYP27B1 (coding for 1α-hydroxylase) expression as well as in serum 1,25(OH)_2_D_3_ levels, compared with mice fed isocaloric glucose- or fructose-normal Ca^2+^ diets. Replacing glucose with fructose prevented these increases in Ca^2+^ transporter, CYP27B1, and 1,25(OH)_2_D_3_ levels induced by a low Ca^2+^ diet. In adult mice fed for three mo a normal Ca^2+^ diet, renal expression of CYP27B1 and of CYP24A1 (24-hydroxylase) decreased and increased, respectively, when the carbohydrate source was fructose instead of glucose or starch. Intestinal and renal Ca^2+^ transporter activity and expression did not vary with dietary carbohydrate. To determine the time course of fructose effects, a high fructose or glucose diet with normal Ca^2+^ levels was fed to adult rats for three mo. Serum levels of 1,25(OH)_2_D_3_ decreased and of FGF23 increased significantly over time. Renal expression of CYP27B1 and serum levels of 1,25(OH)_2_D_3_ still decreased in fructose- compared to those in glucose-fed rats after three mo. Serum parathyroid hormone, Ca^2+^ and phosphate levels were normal and independent of dietary sugar as well as time of feeding. Thus, chronically high fructose intakes can decrease serum levels of 1,25(OH)_2_D_3_ in adult rodents experiencing no Ca^2+^ stress and fed sufficient levels of dietary Ca^2+^. This finding is highly significant because fructose constitutes a substantial portion of the average diet of Americans already deficient in vitamin D.

## Introduction

Fructose is one of the key components of the American diet and represents more than 10% of daily calorie intake [1]. This remarkable and recent increase in fructose consumption coincides with an equally striking increase in prevalence of vitamin D deficiency or insufficiency in developed countries [2]. Since excessive fructose consumption and vitamin D deficiency are each associated with similar metabolic diseases (i.e., hypertension, chronic kidney disease, metabolic syndrome, obesity) [3], [4], [5], a better understanding of the interaction between fructose and vitamin D metabolism will contribute to better health recommendations. Fructose absorption across the intestinal apical membrane is mediated by the facilitative glucose transporter GLUT5 while basolateral exit is mediated by GLUT2 [6], [7]. Fructose is then metabolized in the liver and to a lesser extent in the kidney.

In the event of decreased Ca^2+^ levels in the serum due to low dietary intake or increased demand of Ca^2+^, the synthesis of the hormonally active form of vitamin D, 1,25-dihydroxyvitamin D_3_ (1,25(OH)_2_D_3_) is enhanced, leading to adaptive increases in intestinal Ca^2+^ absorption. If normal serum Ca^2+^ level cannot be maintained by intestinal absorption, then 1,25(OH)_2_D_3_ together with the parathyroid hormone (PTH) will mobilize bone Ca^2+^ and increase reabsorption of Ca^2+^ from the renal distal tubule [8]. Active intestinal and renal Ca^2+^ transport involves Ca^2+^ entry through the apical Ca^2+^ channel transient receptor potential vanilloid 6 and 5 (TRPV6 and TRPV5) respectively, its intracellular diffusion via Ca^2+^-binding proteins (CaBP9k and CaBP28k respectively), and its extrusion across the basolateral membrane mainly through the plasma membrane Ca^2+^-ATPase (PMCA1). 1,25(OH)_2_D_3_ stimulates the transcription, via the vitamin D receptor (VDR) [9], of TRPV6 and TRPV5, as well as CaBP9k and CaBP28k.

Our recent work has demonstrated that excessive fructose consumption affects 1,25(OH)_2_D_3_ and Ca^2+^ metabolism [10], [11]. In both lactating and rapidly growing weanling rats, fructose consumption inhibits adaptive increases in intestinal and renal Ca^2+^ transport, mainly by preventing the upregulation of TRPV6 and CaBP9k expression in the duodenum, and to a lesser extent, of TRPV5 and CaBP28k in the kidney. The mechanism underlying its inhibitory effect on Ca^2+^ transport is that chronic fructose intake also inhibits the adaptive increases in synthesis and blood levels of 1,25(OH)_2_D_3_ regulating intestinal and renal Ca^2+^ transport systems. Highlighting the key role of 1,25(OH)_2_D_3_, treatments with 1,25(OH)_2_D_3_ rescued the inhibitory effect of chronic fructose intake on intestinal Ca^2+^ absorption in weanling rats [11].

The precursor vitamin D_3_ (originating from the skin or diet) is initially hydroxylated by 25-hydroxylase (encoded by *CYP2R1*) in the liver, producing calcidiol or 25(OH)D_3_ that is further hydroxylated by 1α-hydroxylase (encoded by *CYP27B1*) to 1,25(OH)_2_D_3_ in the kidney. The circulating level of 1,25(OH)_2_D_3_ is balanced between synthesis by 1α-hydroxylase and degradation by 24-hydroxylase (encoded by *CYP24A1*), also mainly expressed in the kidney [12]. When serum levels of Ca^2+^ and 1,25(OH)_2_D_3_ are normal, 1,25(OH)_2_D_3_ inhibits its own synthesis by reducing CYP27B1 expression, while low serum levels of 1,25(OH)_2_D_3_ are often associated with a compensatory increase in CYP27B1 expression to restore homeostasis [13]. Chronic fructose-feeding seems to disrupt this balance in rats undergoing a Ca^2+^ challenge. In fructose-fed lactating rats and in fructose-fed rat models of chronic kidney disease, both 1,25(OH)_2_D_3_ levels and CYP27B1 expression are low [10], [11]. In rapidly growing, postweaning rats, fructose feeding not only reduced 1,25(OH)_2_D_3_ and CYP27B1 levels, but also increased CYP24A1 expression, suggesting that high fructose intakes enhance the catabolism and impair the synthesis of 1,25(OH)_2_D_3_ [11]. However, whether this occurred due to low Ca^2+^ or some other mechanism could not be determined.

The aim of this study is to test the hypothesis that chronic fructose consumption causes 1,25(OH)_2_D_3_ insufficiency independent of its effects on adaptive adjustments in Ca^2+^ transport. We first demonstrated that adult mice challenged with a low Ca^2+^ diet failed to upregulate Ca^2+^ transporter expression if fed a high 43% fructose diet for five wk, because fructose prevented compensatory increases in 1,25(OH)_2_D_3_ levels. To minimize potential diet-induced changes in Ca^2+^ transport, we then fed a second group of adult mice with normal Ca^2+^ but with a much higher (60%) fructose concentration for a longer duration of three mo, and eventually found the excessive fructose consumption did reduce levels of 1,25(OH)_2_D_3_ independent of Ca^2+^ transporter expression. Finally, we used rats to enable us to sample blood levels of 1,25(OH)_2_D_3_ and of other hormones, as well as of Ca^2+^ and Pi concentrations, each month and determine the time course of fructose effects on 1,25(OH)_2_D_3_.

## Materials and Methods

All the procedures in this study were approved by the Institutional Animal Care and Use Committee, Rutgers, The State University of New Jersey.

### Experimental Design

Experimental diets were modified from a standard American Institute of Nutrition (AIN)-93G formula by the manufacturer (Research Diets, New Brunswick, NJ). Previous work indicated that 4 wk is sufficient for fructose consumption to have significant effects on intestinal Ca^2+^ transport and serum 1,25(OH)_2_D_3_ levels [11]. Male mice and rats were used and were kept under standard conditions: 12-h light-dark cycle and 24°C. *Study 1: mice fed fructose for five wk.* Four-wk-old C57BL/6 mice (Jackson Laboratory, Bar Harbor, Maine), of similar initial body weights were randomly divided into four groups (*n* = 5) and pair-fed a 43% glucose or 43% fructose diet each containing normal (0.5%) or deficient Ca^2+^ (0.02%) levels (body weights in **Fig. S1**). The levels of Ca^2+^ in the diet were based on previous studies [14], [15]. The rationale underlying dietary fructose concentrations is in the discussion, under “Limitations”. *Study 2: mice fed fructose for three mo.* Four-wk-old mice were randomly divided into three groups (*n* = 5) and fed *ad libitum* a 63% glucose, 63% fructose, or 63% starch diet containing normal Ca^2+^ levels for three mo (body weights in **Fig. S2**). The higher dietary fructose concentration and the longer feeding duration were designed to attempt to determine fructose effects on 1,25(OH)_2_D_3_ under conditions of Ca^2+^ sufficiency. *Study 3: rats fed fructose for three mo.* Four wk old rats were randomly divided into two groups (*n* = 6) fed a 43% glucose or 43% fructose diet containing normal Ca^2+^ levels *ad libitum* for three mo (body weights in **Fig. S3**). Rats were utilized to ensure a large enough volume of serum would be collected each month for monitoring of Ca^2+^, 1,25(OH)_2_D_3_, phosphate (Pi), as well as the putative Pi and 1,25(OH)_2_D_3_ regulators, fibroblast growth factor 23 (FGF23) and parathyroid hormone (PTH).

### *In vitro* Intestinal Ca^2+^ Transport

Intestinal segments were everted immediately after isolation and prepared as sacs to determine Ca^2+^ transport rates at 37°C as described previously [3]. The everted gut sacs were made by using the first 4-cm of proximal duodenum where active transcellular transport of Ca^2+^ is localized [14] and then incubated in Ca^2+^ transport buffer [3]. The *inner* serosal and *outer* luminal compartments had equal initial concentrations (0.25 mM) of nonradioactive Ca^2+^, then a tracer concentration of ^45^Ca^2+^ was added to the outer mucosal compartment. After 1 h, the active accumulation of ^45^Ca^2+^ in the inner serosal compartment was calculated as a ratio of the final concentration of ^45^Ca^2+^ in compartments, inside/outside.

### Serum

Following earlier work [3], serum Pi concentrations were determined using QuantiChrom Pi Assay Kit (BioAssay Systems, Hayward, CA). The total serum Ca^2+^ concentrations were determined by previously described techniques using flame atomic absorption spectrophotometry (Perkin-Elmer Model 633, Norwalk, CT) [16].

### 1,25(OH)_2_D_3_, PTH and FGF23 Assays

Following earlier work [3], serum 1,25(OH)_2_D_3_ levels were measured by enzyme immunoassay (ImmunoDiagnosticSystems (IDS), Arizona). Serum samples were de-lipidated by adding a solution containing dextran sulphate and magnesium chloride, and then centrifuged at 10000×g for 10 min. The resulting supernatant containing the de-lipidated serum is then 1,25(OH)_2_D_3_ immuno-extracted from potential cross-reactants by incubation for 90 minutes with a highly specific solid phase monoclonal anti-1,25(OH)_2_D_3_ before assaying by intact enzyme-linked immuno-sorbent assay (ELISA) for 1,25(OH)_2_D_3_ levels according to the manufacturer’s instructions [17]. Intact FGF23 measurement was performed by ELISAs according to manufacturer’s instructions (Kainos, Tokyo, Japan). PTH was determined using a 2-site sandwich ELISA (Immunotopics, San Clemente, CA, USA).

### Western Blot Analysis

Western blot analyses were performed using 50 μg of intestinal or renal protein extracts following earlier work [3]. For CaBP9k (anti-rabbit, Swant Swiss Antibodies, Switzerland), 4–20% Tris-HCl gels (BioRad, Hercules, CA) were used. For CYP27B1 (anti-rabbit, Santa Cruz Biotechnology Inc., CA) and CYP24A1 (Sigma Chemicals, MO), 12% Tris-HCl gels were used. All membranes were stripped and re-probed with β-actin antibody (anti-mouse, Chemicon International, MA). The membrane was blocked with 5% nonfat milk in Tris-buffered saline-Tween 20 (TBS-T) (0.1% Tween 20, 50 mmol/L Tris-HCL, 137 mmol/L NaCl, pH 7.4) buffer for 1 h. The blots were then incubated with the primary antibody, CaBP9k, CYP24A1 and CYP27B1 diluted 1∶1000, 1∶200 and 1∶1000, respectively, in 5% blocking agent in TBS-T buffer overnight at 4°C. Anti-β-actin antibody was diluted 1∶2000 in the same buffer but incubated with the membrane for one h at room temperature. The membranes were incubated with secondary antibodies, anti-rabbit (1∶20000, GE Healthcare Life Sciences, PA) or anti-mousse (1∶5000, GE Healthcare Life Sciences, PA), for one h at room temperature. The western blot was revealed by enhanced chemiluminescent (ECL) HRP substrate (Thermo Scientific, Rockford, IL USA).

### Real Time RT-PCR

Total RNA from homogenized intestinal mucosa or kidney was isolated, reverse transcribed, and real-time RT-PCR performed using Mx3000P (Stratagene, La Jolla, CA) as previously described [18]. The reference gene was α-elongation factor1 (*EF1α*) whose expression is independent of age and dietary carbohydrate [19]. Previously published primer sequences and annealing temperatures [3] [19] are listed in **Table S1.** To examine the effects of animal age on expression of intestinal Ca^2+^ transporters and of renal 1,25(OH)_2_D_3_ metabolic enzymes, the glucose-fed mouse samples from Studies 1 and 2 were re-run together through real time RT-PCR with results normalized to the older, 4-mo-old, glucose-fed mice of study 2.

### Statistical Analyses

Data are presented as means ± SEM. For studies one and three, a two-way ANOVA was used to determine the effect of fructose and Ca^2+^ level, and of fructose and feeding time course, respectively. If an initial two-way ANOVA indicated a significant effect, a one-way ANOVA followed by LSD test (STATVIEW, Abacus Concepts) determined if differences existed between groups. Differences were considered significant at *P*<0.05. In study two, a one-way ANOVA was used to test for fructose effects.

## Results

### Study 1: Mice Fed Ca^2+^-deficient Diets for Five Weeks

**Figure 1:**
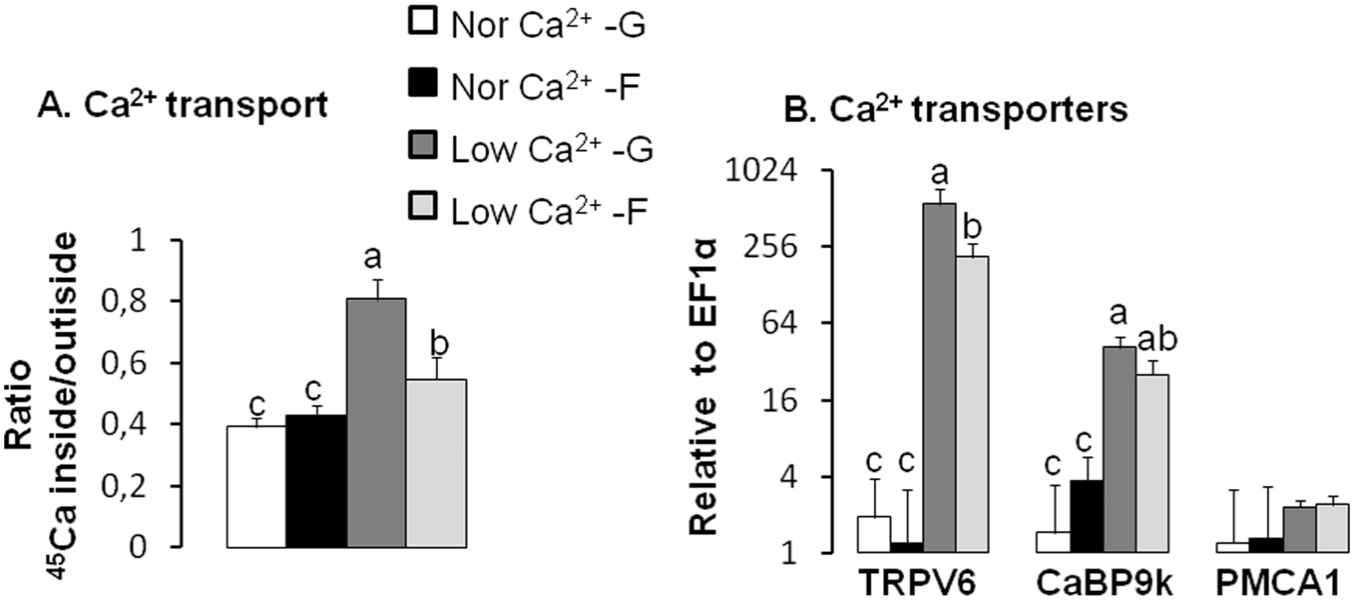
Fructose-fed mice demonstrate an inhibition of compensation for intestinal Ca^2+^transport rate and transporter expression induced by dietary Ca^2+^-deficiency. (A) Ca^2+^ transport was measured using everted duodenal sacs from mice fed diets containing either 43% glucose or fructose, in combination with either normal or low Ca^2+^. Active transepithelial Ca^2+^ transport from the luminal to the basolateral compartment was expressed as a ratio of the final quantity of ^45^Ca^2+^ inside/outside of the everted gut sacs. (B) mRNA expression levels of intestinal Ca^2+^ (TRPV6, CaBP9k, PMCA1) transporters. All expression data were analyzed by real-time PCR using *EF1*α as a reference and then normalized to levels in mice fed a normal Ca^2+^-glucose diet (Nor Ca^2+^-G). Data are means ± SEM (*n* = 6 per group). Differences (*P*<0.05) among means are indicated by differences in superscript letters, as analyzed by 1-way ANOVA LSD. Thus, within a gene of interest, bars with superscript “a” are > bars with “b” which in turn are > bars with “c”. Dietary fructose inhibits compensatory increases in Ca^2+^ transporter activity and expression induced by dietary Ca^2+^-deficiency.

Differences in intestinal Ca^2+^ transport and transporter mRNA were associated with a low Ca^2+^ diet containing fructose as main carbohydrate source. Active transepithelial Ca^2+^ transport rate was modest in mice fed fructose-based, normal Ca^2+^ diet and was similar to that in mice fed a glucose-based, normal Ca^2+^ diet (**Fig. 1A**). When dietary Ca^2+^ level was made deficient, active transepithelial Ca^2+^ transport was enhanced by >2 fold when the carbohydrate source in the diet was glucose. However, no adaptive increases occurred on the fructose-based diet. We investigated the mRNA expression of the three transporters known to mediate active Ca^2+^ transport in the duodenum: TRPV6, CaBP9k and PMCA1 (**Fig. 1B**). mRNA levels of TRPV6 and CaBP9k increased significantly by ∼400- and 30-fold, respectively, in mice fed a glucose-based, low Ca^2+^ diet compared to the glucose-based, normal Ca^2+^ diet, paralleling the low Ca^2+^-induced increase in Ca^2+^ transport. In mice fed a fructose-based, low Ca^2+^ diet these mRNA levels increased by ∼150- and 10-fold, respectively, compared to those fed normal Ca^2+^ diet. Thus, for TRPV6 and CaBP9k, dietary fructose dampened adaptive increases of Ca^2+^ transporter expression by three-fold compared to glucose. Differences in CaBP9k protein levels clearly paralleled those of CaBP9k mRNA (**Fig. S4**). As we previously demonstrated, intestinal GLUT5 expression increased with dietary fructose, regardless of dietary Ca^2+^ level (**Fig. S5**), suggesting that the inhibitory effect of fructose on Ca^2+^ transporter expression is specific.

**Figure 2:**
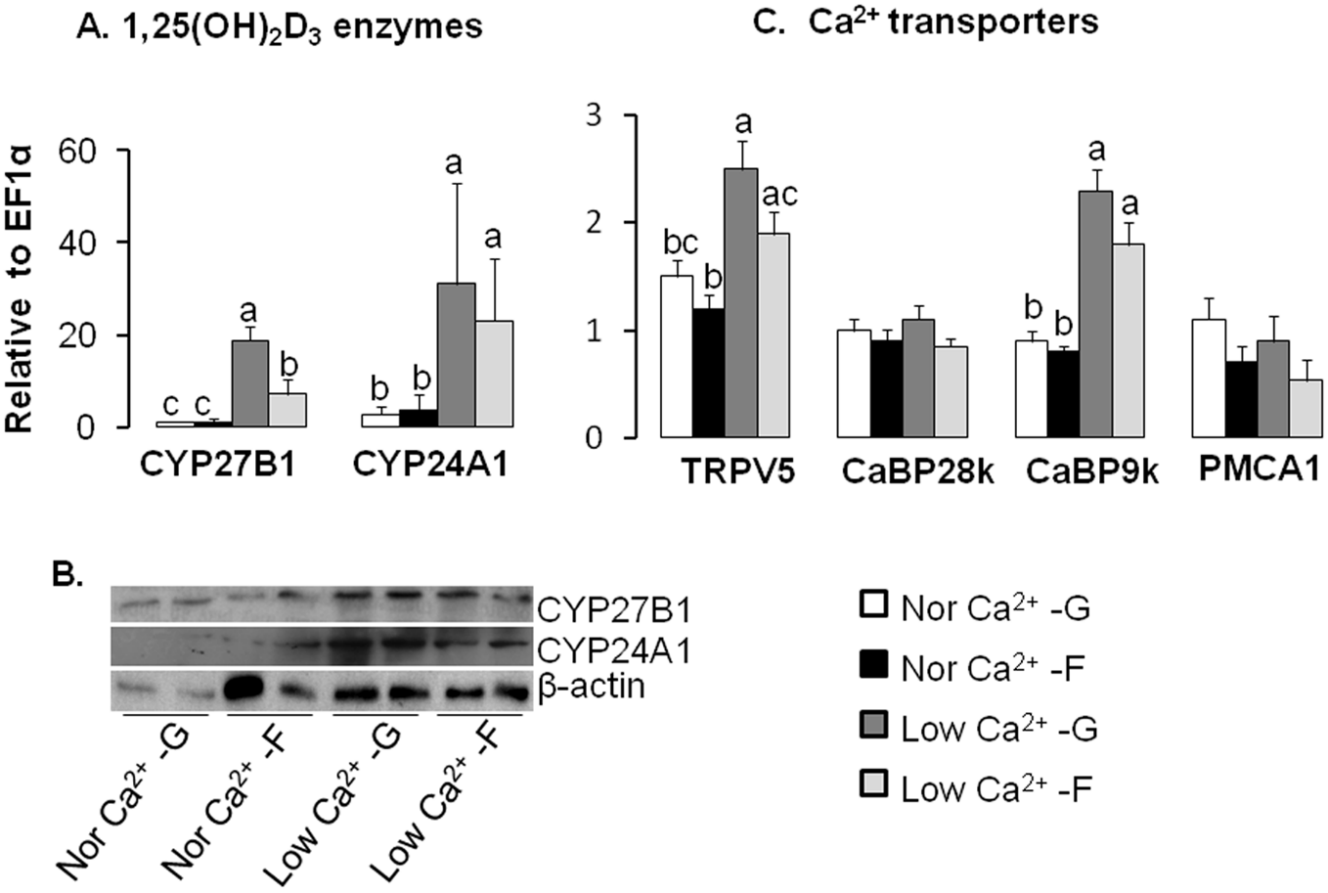
Fructose inhibits compensatory increases in renal expression of the 1,25(OH)_2_D_3_ metabolizing enzyme CYP27B1 induced by dietary Ca^2+^deficiency in mice. (A) mRNA expression levels of renal CYP27B1 (1α-hydroxylase) and CYP24A1 (24-hydroxylase). (B) The protein abundance of CYP27B1 and CYP24A1, using β-actin as a reference. (C) mRNA expression levels of renal TRPV6, CaBP28k, CaBP9k and PMCA1. Nor  =  normal; G  =  glucose; F  =  fructose. All mRNA expression level data were analyzed by real-time PCR using *EF1*α as a reference and normalized to levels in mice fed glucose and normal Ca^2+^ diet. Data are means ± SEM (*n* = 5–6 per group). Differences (*P*<0.05) among means are indicated by differences in superscript letters, as analyzed by 1-way ANOVA LSD. Thus, within a gene of interest, bars with superscript “a” are > bars with “b” which in turn > bars with “c”. Dietary fructose inhibits compensatory increases in renal expression of Ca^2+^ transporters and 1,25(OH)_2_D_3_ metabolizing enzymes induced by dietary Ca^2+^-deficiency.

Differences in renal mRNA expression of synthesis and degradation enzymes for 1,25(OH)_2_D_3_ were associated with fructose and low Ca^2+^ diets. In the kidney of mice fed low Ca^2+^, mRNA levels of CYP27B1 increased significantly by 17- and 7-fold, respectively for glucose- and fructose-based diets, compared to a normal Ca^2+^ diet with glucose or fructose as carbohydrate source (**Fig. 2A**). Thus, dietary fructose lead to a significantly lower expression of CYP27B1 when associated with low Ca^2+^ levels, as compared to dietary glucose. CYP24A1 mRNA expression was significantly upregulated by ∼14-fold with low Ca^2+^, regardless of dietary sugar. Similar patterns of expression were found with protein levels (**Fig. 2B**).

As expected, the circulating level of 1,25(OH)_2_D_3_ was significantly increased with low Ca^2+^ (**Table 1**) and paralleled the increases in renal mRNA and protein expression of CYP27B1 and CYP24A1 with low Ca^2+^. However, dietary fructose dampened the low Ca^2+^-induced increase in 1,25(OH)_2_D_3_ level which was not significantly different from those in mice fed normal Ca^2+^ diets (P>0.075 by one-way ANOVA). A low Ca^2+^ diet was associated with a significant 40% decrease in circulating level of FGF23, independent of dietary sugar concentrations. However, serum concentrations of Ca^2+^ and Pi which are primarily regulated by 1,25(OH)_2_D_3_ and FGF23, did not vary with diet.

**Table 1:** Blood chemistry of mice after consuming glucose- or fructose-based low and normal Ca^2**+**^ diets for five weeks.

Calcium level	Normal Ca^2+^		Low Ca^2+^		Two-way ANOVA significance		
*Dietary sugar*	*Glucose*	*Fructose*	*Glucose*	*Fructose*	*Ca^2+^*	*Sugar*	*interaction*
1,25-(OH)_2_D_3_ (pmol/L)	121±17^b^	118±15^b^	626±197^a^	389±44^ab^	0.008	0.236	0.246
FGF23 (pg/mL)	98±4^x^	107±6^x^	63±4^y^	68±8^y^	<0.0001	0.119	0.888
Serum Ca^2+^ (mg/dL)	10.0±0.4	10.4±0.3	9.9±0.1	10.7±0.4	0.351	0.214	0.895
Serum Pi (mM)	1.9±0.2	2.0±0.1	2.0±0.1	2.2±0.2	0.469	0.559	0.926

Normal Ca^2+^  = 0.5%; Low Ca^2+^  = 0.02%; Glucose or Fructose = 43%; FGF  =  fibroblast growth factor. *n* = 5–6 per group. Data are means ± SEM. Means with different superscript letters are significantly different from others in the same row (*P*<0.05 by *posthoc* LSD test).

In the kidney, differences in mRNA expression of Ca^2+^ transporters were associated with low Ca^2+^ diets. Low dietary Ca^2+^ induced a ∼two-fold increase in renal TRVP5 and CaBP9k mRNA levels in combination with either glucose or fructose (**Fig. 2C**). In contrast, CaBP28k and PMCA1 mRNA expression did not vary with diet.

### Study 2: Mice Fed Fructose for Three Months

**Figure 3:**
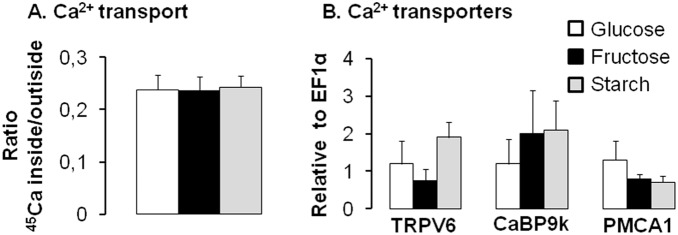
Chronic consumption of fructose has no significant effect on intestinal Ca^2+^ transport rate and transporter mRNA expression in mice fed for three mo a high fructose, starch, or glucose diet containing normal Ca^2+^ levels. (A) Active transduodenal Ca^2+^ transport from the luminal to the basolateral compartment was expressed as a ratio of the final quantity of (^45^Ca^2+^ inside/^45^Ca^2+^ outside) of the everted sacs of mice fed diets containing 63% fructose, glucose or starch. (B) mRNA expression levels of intestinal Ca^2+^ (TRPV6, CaBP9k, PMCA1) transporters. All expression data were analyzed by real-time PCR using *EF1*α as a reference and normalized relative to levels seen in mice fed glucose diet. Data are means ± SEM (*n* = 5 per group). Chronic consumption of high fructose levels has no significant effect on intestinal Ca^2+^ transport rate and transporter mRNA expression.

In order to determine if chronic high fructose intake alters CYP27B1 and CYP24A1 expression independently of Ca^2+^demand and metabolism, mice were challenged for three mo with diets containing higher levels of fructose and sufficient levels of Ca^2+^. Similar rates of food intake were observed among mice fed glucose, fructose or starch diets (average of 0.09±0.01 g per g of bw per day for all mice). Duodenal Ca^2+^ transport rates did not vary with carbohydrate source and were similar in glucose-, fructose- and starch-fed mice (**Fig. 3A**). TRPV6, CaBP9k and PMCA1 mRNA expression did not vary among the three diets (**Fig. 3B**). CaBP9k protein expression was not detected by Western blot (data not shown), indicating a low expression level in the 4 mo old mice fed sufficient dietary Ca^2+^.

**Figure 4:**
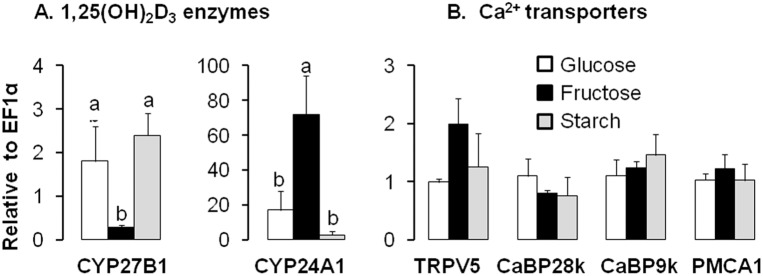
Expression of 1,25(OH)_2_D_3_ metabolizing enzymes and of Ca^2+^transporters in the kidney of mice fed for three mo a high fructose, starch, or glucose diet containing normal Ca^2+^ levels. (A) Marked effects of excessive fructose intake on mRNA expression levels of renal 1,25(OH)_2_D_3_ metabolic enzymes, CYP27B1 and CYP24A1. (B) mRNA expression levels of renal TRPV6, CaBP28k, CaBP9k and PMCA1. All expression data were analyzed by real-time PCR using *EF1*α as a reference and normalized relative to levels seen in mice fed glucose diet. Data are means ± SEM (*n* = 5 per group). Differences (*P*<0.05) among means are indicated by differences in superscript letters, as analyzed by 1-way ANOVA LSD. Thus, within a gene of interest, bars with superscript “a” are > bars with “b”. Chronic consumption of high fructose levels has dramatic effects on mRNA expression of renal 1,25(OH)_2_D_3_ metabolizing enzymes but not on Ca^2+^ transporter mRNA expression.

In the kidney, fructose feeding had remarkable effects on the mRNA expression of genes involved in regulating 1,25(OH)_2_D_3_ levels. CYP27B1 expression decreased and CYP24A1 expression increased when compared to starch or glucose feeding (**Fig. 4A**). However, fructose had no effect on TRPV5, CaBP9k, CaBP28k, or PMCA1 expression (**Fig. 4B**). Taken together, these findings from intestine and kidney indicate that excessive fructose consumption may regulate 1,25(OH)_2_D_3_ independent of any effect on Ca^2+^ transport.

**Figure 5:**
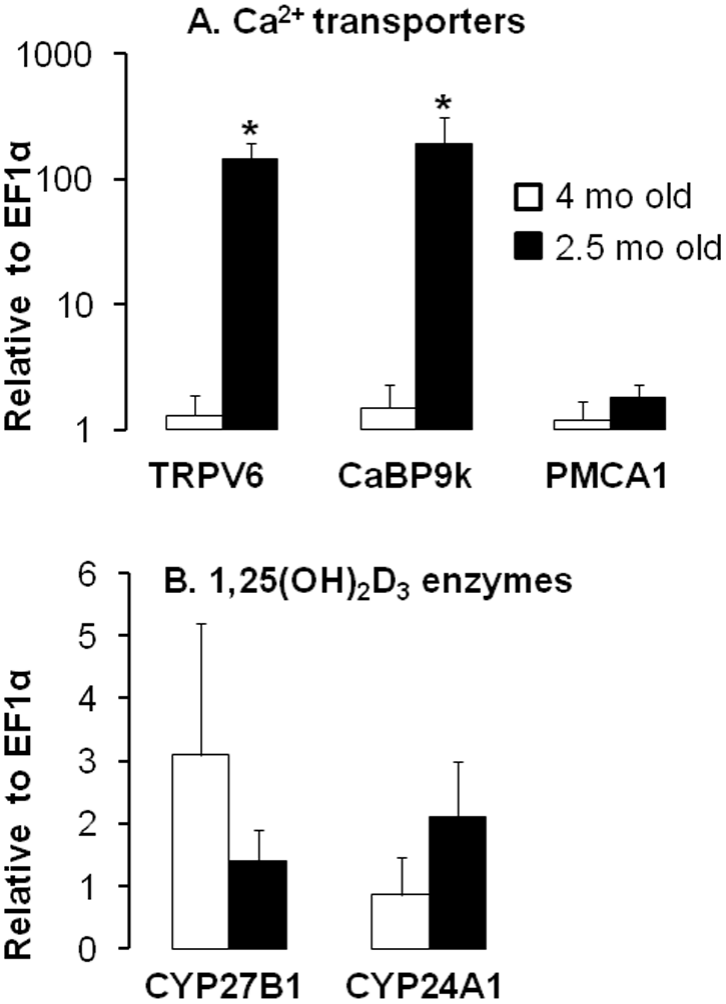
Effects of age on expression of intestinal Ca^2+^ transporters and of renal 1,25(OH)_2_D_3_ metabolic enzymes in mice. A comparison of the expression levels of Ca^2+^ transporters (**A**) and 1,25(OH)_2_D_3_ metabolic enzymes (**B**) in four mo old mice after three mo of feeding on normal Ca^2+^ diet containing 63% glucose (from study 2) and in two mo old mice fed a normal Ca^2+^ diet containing 43% glucose for five wk (from study 1). All expression data were analyzed by real-time PCR using *EF1*α as a reference and normalized relative to levels seen in four mo old mice. Data are means ± SEM (*n* = 5–6 per group). Differences (*P*<0.05) between means are indicated by asterisks. Expression of intestinal Ca^2+^ transporters decreases with age.

To examine possible mechanisms behind the difference in Ca^2+^ transport rates between Figs. 1A and 3A, we reanalyzed the intestinal transporter and renal 1,25(OH)_2_D_3_ regulating enzymes in a direct comparison of mRNA expression levels in mice fed normal Ca^2+^ and glucose from study 1 (those mice were 2.5 mo old at sacrifice) and mice fed normal Ca^2+^ and glucose from study 2 (those mice were 4 mo old at sacrifice). Intestinal mRNA expression levels of TRPV6 and CaBP9k were both ∼100-fold lower at 4 than at 2.5 mo of age (**Fig. 5A** **)**. Low CaBP9k expression at 4 mo-old mice fed normal Ca^2+^ explains the undetectable CaBP9k protein levels in these mice. Despite the large age-related differences in Ca^2+^ transport and transporter expression, expression of renal CYP27B1 and CYP24A1 was the same (**Fig. 5B**).

### Study 3: Rats Fed Fructose for Three Months

**Figure 6:**
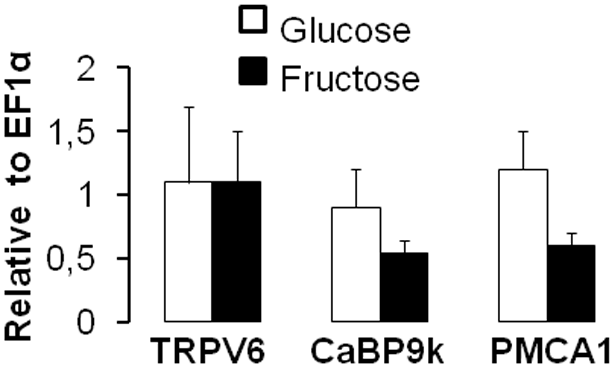
Effect of chronic fructose feeding on Ca^2+^ transporter expression in rat intestine. Rats were fed for three mo a normal Ca^2+^ diet containing either 43% glucose or fructose. mRNA expression levels of intestinal Ca^2+^ (TRPV6, CaBP9k, PMCA1) transporters remained similar between diets.

**Figure 7:**
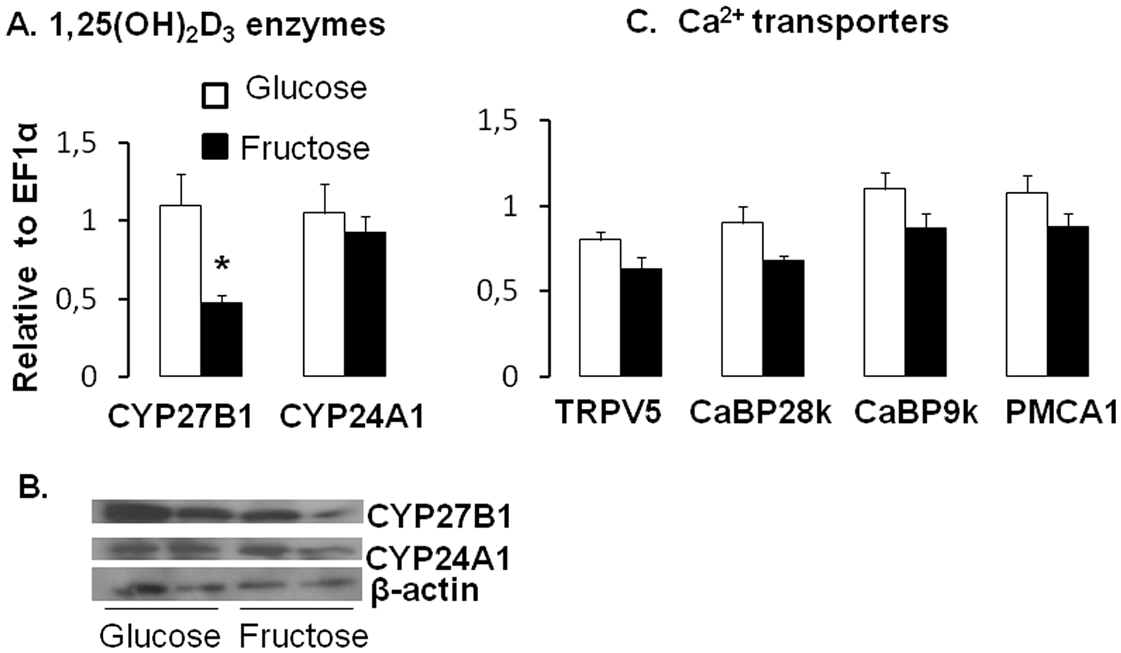
Effect of chronic fructose feeding on renal expression of 1,25(OH)_2_D_3_ metabolizing enzymes and of Ca^2+^transporters. Rats were fed for three mo a normal Ca^2+^ diet containing either 43% glucose or fructose. (**A**) mRNA expression level of renal 1,25(OH)_2_D_3_ metabolic enzymes, CYP27B1 and CYP24A1 (**B**) The protein abundance of CYP27B1 and CYP24B1 using β-actin as a reference. (**C**) mRNA expression levels of renal TRPV6, CaBP28k, CaBP9k and PMCA1. All expression data were analyzed by real-time PCR using *EF1*α as a reference and normalized relative to levels seen in rat fed glucose diet. Data are means ± SEM (*n* = 6 per group). Differences (*P*<0.05) between means are indicated by asterisks. Chronic consumption of high fructose diets reduces mRNA and protein expression of CYP27B1.

Having demonstrated that prolonged feeding of a fructose diet altered renal expression of 1,25(OH)_2_D_3_ metabolizing enzymes independent of changes in expression of Ca^2+^ transporters and binding proteins, we proceeded to evaluate the time course of the fructose effect on 1,25(OH)_2_D_3_ levels. Rats fed a fructose or glucose diet displayed similar rates of food intake as well as similar final body weights (**Fig. S3A, B**). Similar to Study 2, the expression levels of intestinal and renal transporters with normal Ca^2+^ were also the same on fructose- and glucose-based diets (**Figs. 6** **,** **7**). While it might have been possible to get a statistically significant fructose effect with a greater *n*, another reason we failed to get significance was the known age-related reductions in expression of Ca transporters that would also reduce the magnitude of the fructose effect. However, fructose feeding did lead to a two-fold decrease in mRNA expression of the 1,25(OH)_2_D_3_ synthesizing renal enzyme, CYP27B1 (**Fig. 7A**). Meanwhile, CYP24A1 expression did not change. Protein levels demonstrated similar results (**Fig. 7B**).

As expected, the decreased CYP27B1 expression after three mo of consuming a high fructose diet was associated with a significantly lower circulating level of 1,25(OH)_2_D_3_ versus the glucose diet group (**Table 2**). The most pronounced effect was with duration as in both diet groups, 1,25(OH)_2_D_3_ serum concentrations fell by over 40% from two to three and four mo of age. Conversely, circulating levels of FGF23 were not affected by the dietary sugar, and increased about 20% between three and four mo of age, suggesting that age-related reductions in 1,25(OH)_2_D_3_ levels occur prior to increases in FGF23. Serum PTH, Ca^2+^ and Pi did not vary with duration or dietary sugar.

**Table 2:** Time course of the fructose-induced reduction in serum levels of 1,25-(OH)_2_D_3_ in adult rats fed for three mo a normal Ca^2+^ diet with either glucose or fructose.

Feeding duration	one		two		three				
(mo) *Age of rat (mo)*	*two*		*three*		*four*		Two-way ANOVA significance		
Dietary Sugar	Glucose	Fructose	Glucose	Fructose	Glucose	Fructose	*Age*	*Sugar*	*interaction*
1,25-(OH)_2_D_3_ (pmol/L)	358±38^a^	347±27^a^	193±23^bc^	226±19^b^	210±18^b^	131±23^c^	<0.0001	0.398	0.129
FGF23 (pg/mL)	401±37^x^	482±49^x^	390±21^x^	425±57^x^	556±50^y^	537±34^y^	0.008	0.373	0.532
PTH (pg/mL)	333±79	302±34	258±66	256±17	320±64	432±69	0.24	0.67	0.500
Serum Ca^2+^ (mg/dL)	10.4±0.1	10.4±0.1	11.0±0.2	10.7±0.2	11.1±0.6	10.8±0.2	0.281	0.901	0.794
Serum Pi (mM)	1.19±0.03	1.30±0.06	1.31±0.05	1.29±0.06	1.29±0.09	1.46±0.10	0.172	0.1.22	0.373

Glucose or Fructose = 43%. FGF  =  fibroblast growth factor, PTH  =  parathyroid hormone. *n* = 6 per group. Data are means ± SEM. Means with different superscript letters are significantly different from others in the same row (*P*<0.05 by *posthoc* LSD test).

## Discussion

We show in mice that excessive fructose intake inhibits adaptive increases in intestinal Ca^2+^ absorption and in 1,25(OH)_2_D_3_ levels, in response to dietary Ca^2+^ restriction. Thus, regardless of the source of Ca^2+^ challenge (growth [11], lactation [10], renal disease [3], dietary insufficiency (this paper)), excessive fructose consumption will diminish, if not completely abolish, the main physiological responses: increased intestinal absorption and renal reabsorption of Ca^2+^. Very importantly, we also demonstrate in both rats and mice that chronic fructose intake can decrease 1,25(OH)_2_D_3_ levels independent of Ca^2+^ demand, and can alter CYP27B1 or CYP24A1 expression in the absence of physiological and nutritional Ca^2+^ challenge.

### Excessive Fructose Intake Decreases 1,25(OH)_2_D_3_

Results from these current studies and our previous work [10], [11] have consistently shown that fructose feeding prevents adaptive increases in serum levels of 1,25(OH)_2_D_3._ This fructose-induced decrease in serum 1,25(OH)_2_D_3_ concentrations was not associated with a reduction in serum levels of its precursor 25(OH)D_3_, suggesting fructose had little effect on the initial steps of vitamin D synthesis. Instead, these findings indicate a specific effect of fructose at the renal step of either 1,25(OH)_2_D_3_ synthesis or degradation [10]. The fructose-induced decrease in 1,25(OH)_2_D_3_ levels was always associated with a fructose-induced decrease in CYP27B1 expression and less consistently with increased CYP24A1 expression. So far the effect of fructose on 1,25(OH)_2_D_3_ had been observed only when Ca^2+^ had been challenged. We demonstrate in the current study that chronic fructose intake reduces over time normal circulating levels of 1,25(OH)_2_D_3_ independently of any increase in Ca^2+^ requirement.

### Chronic Fructose Intake and Intestinal Ca^2+^ Transport

Total intestinal Ca^2+^ absorption consists of a passive paracellular and a 1,25(OH)_2_D_3_-dependent active transcellular pathway [20]. Since the ultimate source of Ca^2+^ is intestinal absorption, vertebrates undergoing Ca^2+^ stress adaptively increase active Ca^2+^ transcellular transport, achieved by augmenting levels of 1,25(OH)_2_D_3_. Here we confirmed in mouse our previous findings in rat models showing that marked increases in active absorption of Ca^2+^ are associated with equally remarkable increases in TRPV6 and CaBP9K expression, and that high fructose intakes dramatically compromise these adaptive increases in Ca^2+^ transport rates as well as in TRPV6 and CaBP9k mRNA or protein levels [10], [11].

In the present work, under Ca^2+^ restricted conditions, the inhibition of adaptive increases in TRPV6 and CaBP9k expression by fructose likely explains the limited upregulation of active intestinal Ca^2+^transport in rodents with high fructose intakes. CaBP9k and TRPV6 had been shown to play a major role in the adaptation of intestinal Ca^2+^ transport under low Ca^2+^ conditions, and after induction by 1,25(OH)_2_D_3_ [14]. When dietary supply of Ca^2+^ is normal, TRPV6 null mice or CaBP9k null mice display a normal Ca^2+^ transport. However, in double KO TRPV6^−/−^/CaBP9k^−/−^ mice, the adaptive increase in intestinal active Ca^2+^ transport in response to low Ca^2+^ diet or 1,25(OH)_2_D_3_ injection was reduced by 50% compared to that of wild-type mice, indicating that both CaBP9k and TRPV6 are needed for optimal Ca^2+^ transport under a Ca^2+^ challenge condition [14]. The same adaptive increase was also seriously impaired in the single KO TRPV6^−/−^ mice [14], suggesting that TRPV6 plays a major role in active intestinal Ca^2+^ absorption.

We [10], [11] have not found rodent PMCA1 levels to vary with Ca^2+^ stress and other workers have even used this gene for reference or “housekeeping” purposes [21] because of its stable expression even as TRPV6 levels change markedly. This difference in regulation between TRPV’s and PMCA’s is understandable because Ca^2+^ transport across the apical membrane is the rate-limiting step [22].

### Effect of Age

In four mo old mice and rats, fructose did not affect the active Ca^2+^ transport or Ca^2+^ transporter expression likely because active Ca^2+^ transport constitutes a very small component of total intestinal transport in older, Ca^2+^-sufficient animals. Comparing Fig. 3A and 1A, Ca^2+^ transport rate was also lower in older mice (study 2) which were fed normal Ca^2+^ for three mo than in younger mice fed the same sufficient level of dietary Ca^2+^ in Study 1. Compared to the 2.5 mo old mice, the modest rates of active Ca^2+^ transport rates exhibited by four mo old mice were also associated with reduced expression of TRPV6 and CaBP9K, as previously demonstrated in rats [23]. In turn, age-related decreases in Ca^2+^ transport and transporter expression occurring in adult rodents between two and four mo of age may be linked to the age-related decrease in serum 1,25(OH)_2_D_3_ levels occurring simultaneously, as shown in this study and as previously demonstrated in rats [24]. The role of age in modulating 1,25(OH)_2_D_3_ levels should be further investigated in mice on the same diets as a limitation of our age comparison is that different (43% carbohydrate, Study 1 and 63%, Study 2) diets were utilized.

In this and in previous work, adult rats and mice fed sufficient Ca^2+^ and Pi levels and not under Ca^2+^ stress have normal serum Ca^2+^ and Pi levels even when fed high fructose diets. The active, NaPi2b-mediated component of intestinal Pi transport also decreases dramatically in postweaning rodents as growth rate decreases markedly with age [25]. These findings suggest that paracellular transport of Ca^2+^ and Pi may be sufficient to maintain serum levels as demand for these minerals in older rodents is low.

### Interactions among Blood Levels of 1,25(OH)_2_D_3_, FGF23 and PTH

FGF23 is a hormone synthesized mainly in the osteocytes and osteoblasts of bone and regulates blood levels of Pi via removal of Pi transporters from the apical membrane of kidney cells, thus reducing Pi reabsorption and increasing urinary excretion [10], [26]. FGF23 also regulates 1,25(OH)_2_D_3_ by inhibiting CYP27B1 expression and increasing CYP24A1 expression [27]. Moreover, FGF23 synthesis is positively regulated by increased serum levels of 1,25(OH)_2_D_3_ or of Pi.

In a previous study using growing rats, we found that chronic consumption of fructose was associated with a significant increase in circulating levels of FGF23, which could potentially be the mechanism by which fructose reduces 1,25(OH)_2_D_3_ levels [11]. However, in the present study, in mice fed for five wk a fructose-based, low Ca^2+^ diet and in rats fed for three mo a fructose-based, normal Ca^2+^ diet (high-fructose conditions that reduce circulating levels of 1,25(OH)_2_D_3_), FGF23 production did not increase with fructose feeding. In mice, the failure of fructose to induce FGF23 production may be due to the confounding effects of hypocalcaemia that acutely reduces circulating level of FGF23 [28]. Our data confirm this since in Ca^2+^ deficiency, circulating levels of FGF23 are low, thus allowing 1,25(OH)_2_D_3_ circulating levels to remain elevated and prevent decreases in blood Ca^2+^. In adult rats, the mechanism of the fructose-induced decrease in 1,25(OH)_2_D_3_ levels remains unclear, as fructose-induced increases in FGF23 levels were not significant perhaps due to a statistically insufficient number of samples. Fructose, however, is known to injure the kidneys [3], [10], [29] which are the site of 1,25(OH)_2_D_3_ synthesis. PTH levels were not affected by fructose as shown in this as well as in our previous studies [10], [11].

### Limitations

There are limitations to this study, both in conduct and extrapolation of the results to the human condition. Despite similar food intakes, fructose feeding was associated with a significantly greater kidney mass index (kidney weight/body weight) in both the longer-term studies (two and three) in both mice and rats (**Figs. S2 and S3**). Additionally, rodents and humans have different diets. The AIN recommends either 73% (AIN-93M (maintenance) diet) or 63% (AIN-93G (growth) diet) carbohydrate, levels higher than the average for humans (54%) consuming a high carbohydrate diet [30]. Thus, rodents consuming 43 or 63% sugars, though consuming more % carbohydrate than humans, are eating less than the carbohydrate amount and concentration in a standard AIN-93M laboratory rodent diet. The popularity of>60% fructose diets is demonstrated in the fact that >130 published studies in the last five years specifically used pellets containing ∼60% fructose, because such a level typically causes pathological effects in a mouse within 4 wk. Here we used high concentrations of fructose to allow us to detect any potential effect it may have on expected outcomes. While our previous studies found 63% fructose diets to cause a significant decrease in serum 1,25(OH)_2_D_3_ levels [10], [11] after 4–6 wk of feeding, the present paper demonstrated that lowering the amount of fructose in the diet could lead to similar decreases in serum 1,25(OH)_2_D_3_, but only after a longer period of feeding (3 mo). Humans consume lower concentrations of fructose that are often mixed with other carbohydrates, but studies have shown that mixing with glucose may accelerate fructose effects. Because of the cumulative effects of fructose demonstrated here, lower fructose concentrations will likely cause similar deleterious effects as higher fructose concentrations if consumed for a longer time period [31], [32].

### Conclusion and Perspective

Despite these limitations, we have clearly demonstrated that a chronic intake of high levels of dietary fructose can lead to a decrease in circulating levels of 1,25(OH)_2_D_3_ independent of dietary Ca^2+^ levels and of physiological increases in Ca^2+^ requirement. This work is highly relevant since fructose, a sugar contained in many types of foods that are being consumed at high levels, may contribute to the increasing prevalence of vitamin D insufficiency, especially in sensitive populations with high, processed sugar intakes such as in children and teenagers [33], [34]. Indeed, when consumption is normalized to body weight, these age groups are the highest consumers of fructose, with an average total ingestion reaching ∼2 g kg^−1^ day^−1^ occurring at a time when there is a very high demand for vitamin D during growth [35].

## Supporting Information

Figure S1 **The body and kidney weight of mice pair-fed diets containing either 43% glucose or fructose, in combination with either normal or low Ca^2+^. A**) Initial and final body weight after 5 wk of feeding on the experimental diets. **B**) Kidney weight after 5 wk. **C**) Kidney somatic index. Bars are means ± SEM; *n* = 5–8. Nor  =  normal; G  =  glucose; F  =  fructose. Differences (*P*<0.05) among means are indicated by differences in superscript letters, as analyzed by 1-way ANOVA LSD. Thus, bars with superscript “a” are > bars with “b” and similar to those with “ab”.
(TIF)

Figure S2 **The body weight, feeding rate, and kidney weight of mice fed diets containing normal Ca^2+^ and 63% glucose, fructose or starch. A**) Body weight after 3 mo of feeding on the special diets. **B**) Feeding rate per day normalized to body weight. **C**) Kidney weight after 3 mo of feeding on the special diets. **D**) Kidney somatic index. Bars are means ± SEM; *n* = 5–8. Differences (*P*<0.05) among means are indicated by differences in superscript letters, as analyzed by 1-way ANOVA LSD. Thus, bars with superscript “a” are > bars with “b”.
(TIF)

Figure S3 **The body and kidney weight of rat fed diets containing either 43% glucose or fructose. A**) Body weight after 3 mo of feeding of the special diets. **B**) Feeding rate per day normalized to body weight. **C**) Kidney weight after 3 mo of feeding of the special diets. **D**) Kidney somatic index. Bars are means ± SEM; *n* = 5–8. Differences (*P*<0.05) among means are indicated by differences in superscript letters, as analyzed by 1-way ANOVA LSD. Thus, bars with superscript “a” are > bars with “b”.
(TIF)

Figure S4 **CaBP9k protein abundance.** The protein abundance of CaBP9k was determined in the small intestine of mice fed diets containing either 43% glucose or fructose, in combination with either normal or low Ca^2+^. β-actin was used as a reference. Nor  =  normal; G  =  glucose; F  =  fructose.
(TIF)

Figure S5 **mRNA levels of GLUT5.** Expression level of GLUT5 in the intestine of **A**) mice fed diets containing either 43% glucose or fructose, in combination with either normal or low Ca^2+^ for 5 wk; **B**) mice fed diets containing normal Ca^2+^ in combination with 63% glucose, fructose or starch for 3 mo; and **C**) rats fed diets containing either 43% glucose or fructose for 3 mo. All expression data were analyzed by real-time PCR using *EF1*α as a reference and normalized relative to levels seen in rat fed glucose diet. Nor  =  normal; G  =  glucose; F  =  fructose. Data are means ± SEM (*n* = 6 per group). Differences (*P*<0.05) among means are indicated by differences in superscript letters, as analyzed by 1-way ANOVA LSD. Thus, for each study, bars with superscript “a” are > bars with “b”.
(TIF)

Table S1 **Primer sequences**.
(DOC)
