# Supplementary figures and images for: Chronic High Fructose Intake Reduces Serum 1,25 (OH)2D3 Levels in Calcium-Sufficient Rodents
Source: PLoS One. 2014 Apr 9;9(4):e93611. doi: 10.1371/journal.pone.0093611 (PMC3981704; doi:10.1371/journal.pone.0093611)

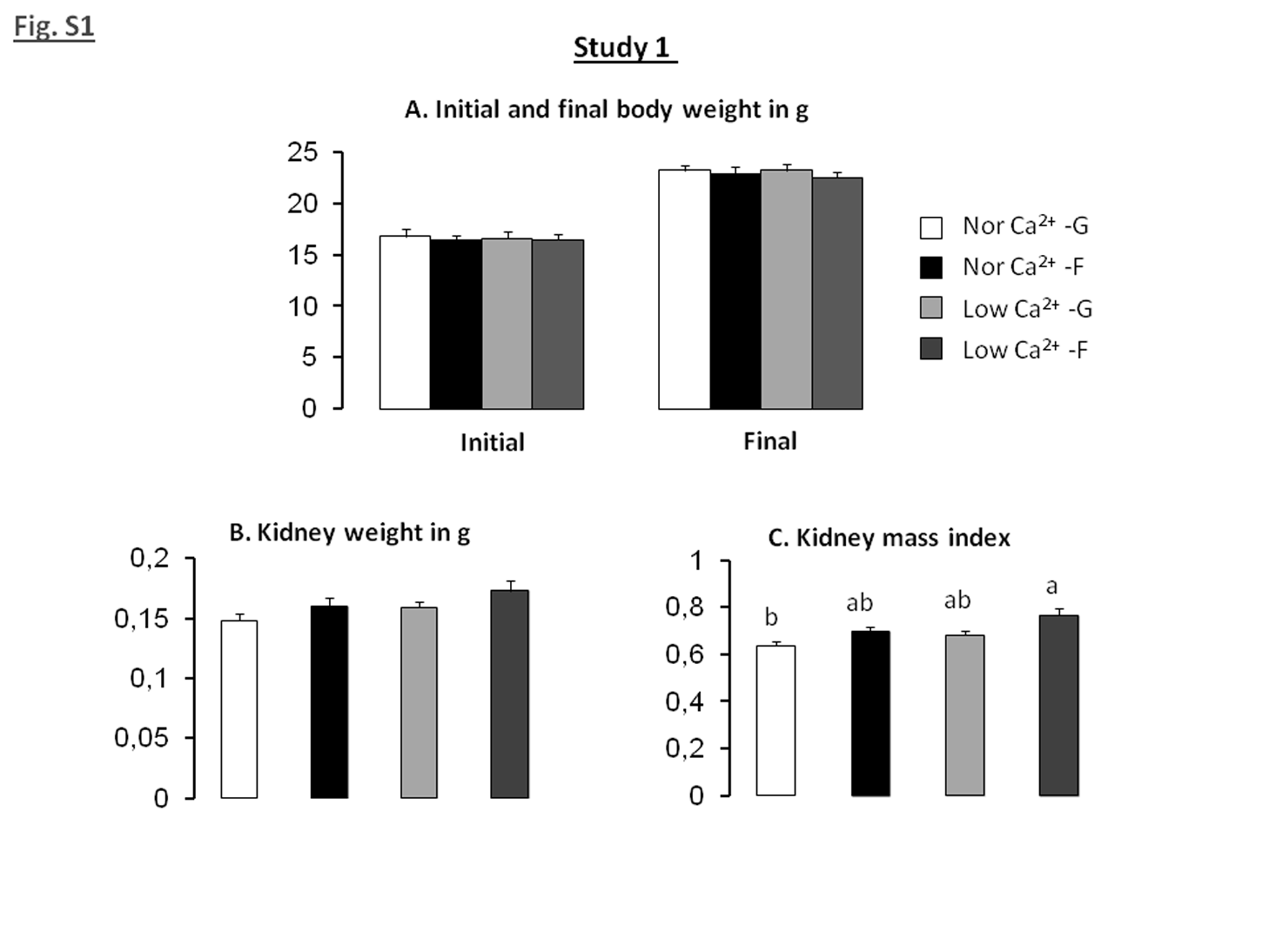

Supplement: Figure S1 — The body and kidney weight of mice pair-fed diets containing either 43% glucose or fructose, in combination with either normal or low Ca2+. A) Initial and final body weight after 5 wk of feeding on the experimental diets. B) Kidney weight after 5 wk. C) Kidney somatic index. Bars are means ± SEM; n = 5–8. Nor = normal; G = glucose; F = fructose. Differences (P<0.05) among means are indicated by differences in superscript letters, as analyzed by 1-way ANOVA LSD. Thus, bars with superscript “a” are > bars with “b” and similar to those with “ab”. (TIF) [file pone.0093611.s001.tif]

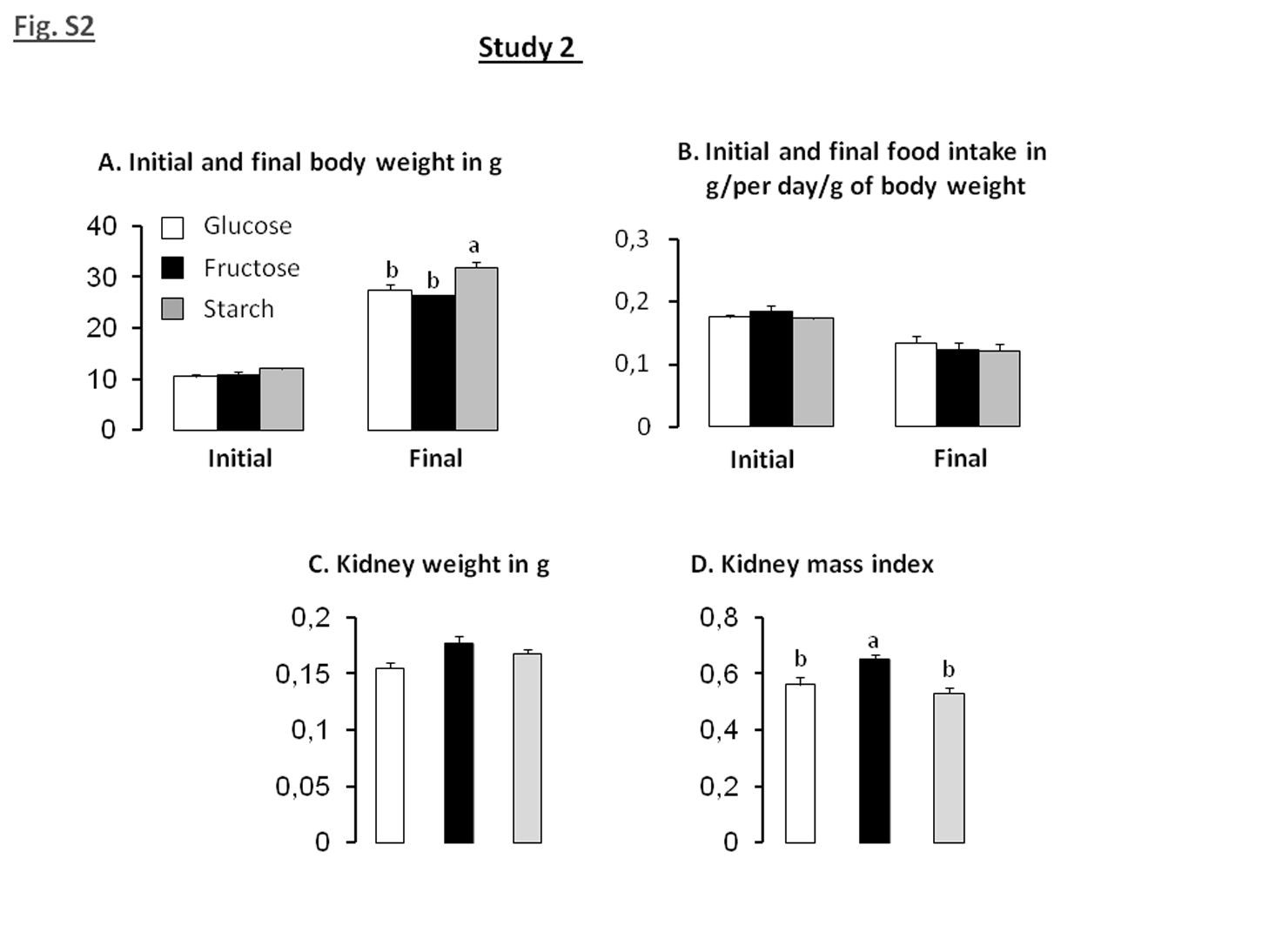

Supplement: Figure S2 — The body weight, feeding rate, and kidney weight of mice fed diets containing normal Ca2+ and 63% glucose, fructose or starch. A) Body weight after 3 mo of feeding on the special diets. B) Feeding rate per day normalized to body weight. C) Kidney weight after 3 mo of feeding on the special diets. D) Kidney somatic index. Bars are means ± SEM; n = 5–8. Differences (P<0.05) among means are indicated by differences in superscript letters, as analyzed by 1-way ANOVA LSD. Thus, bars with superscript “a” are > bars with “b”. (TIF) [file pone.0093611.s002.tif]

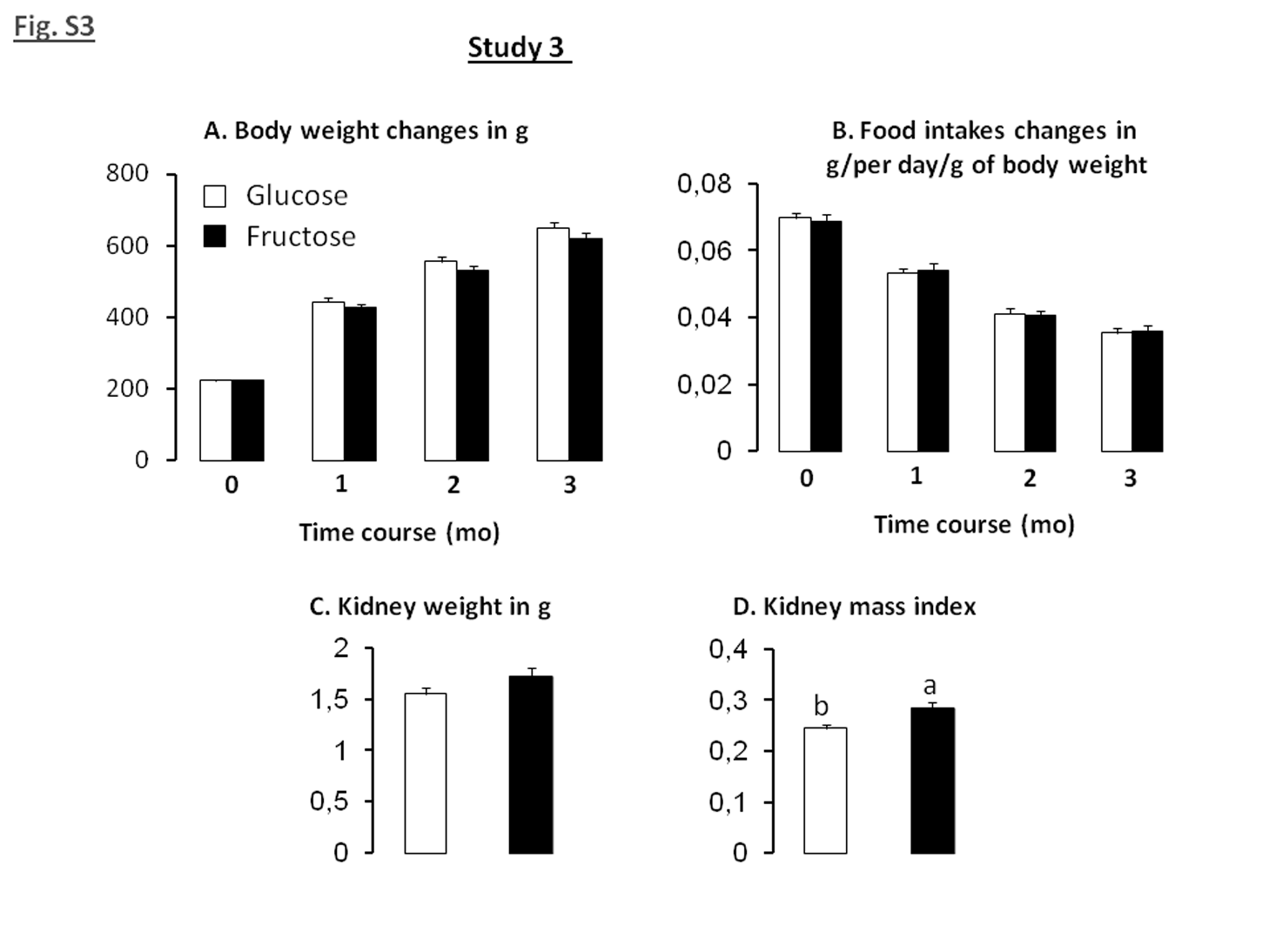

Supplement: Figure S3 — The body and kidney weight of rat fed diets containing either 43% glucose or fructose. A) Body weight after 3 mo of feeding of the special diets. B) Feeding rate per day normalized to body weight. C) Kidney weight after 3 mo of feeding of the special diets. D) Kidney somatic index. Bars are means ± SEM; n = 5–8. Differences (P<0.05) among means are indicated by differences in superscript letters, as analyzed by 1-way ANOVA LSD. Thus, bars with superscript “a” are > bars with “b”. (TIF) [file pone.0093611.s003.tif]

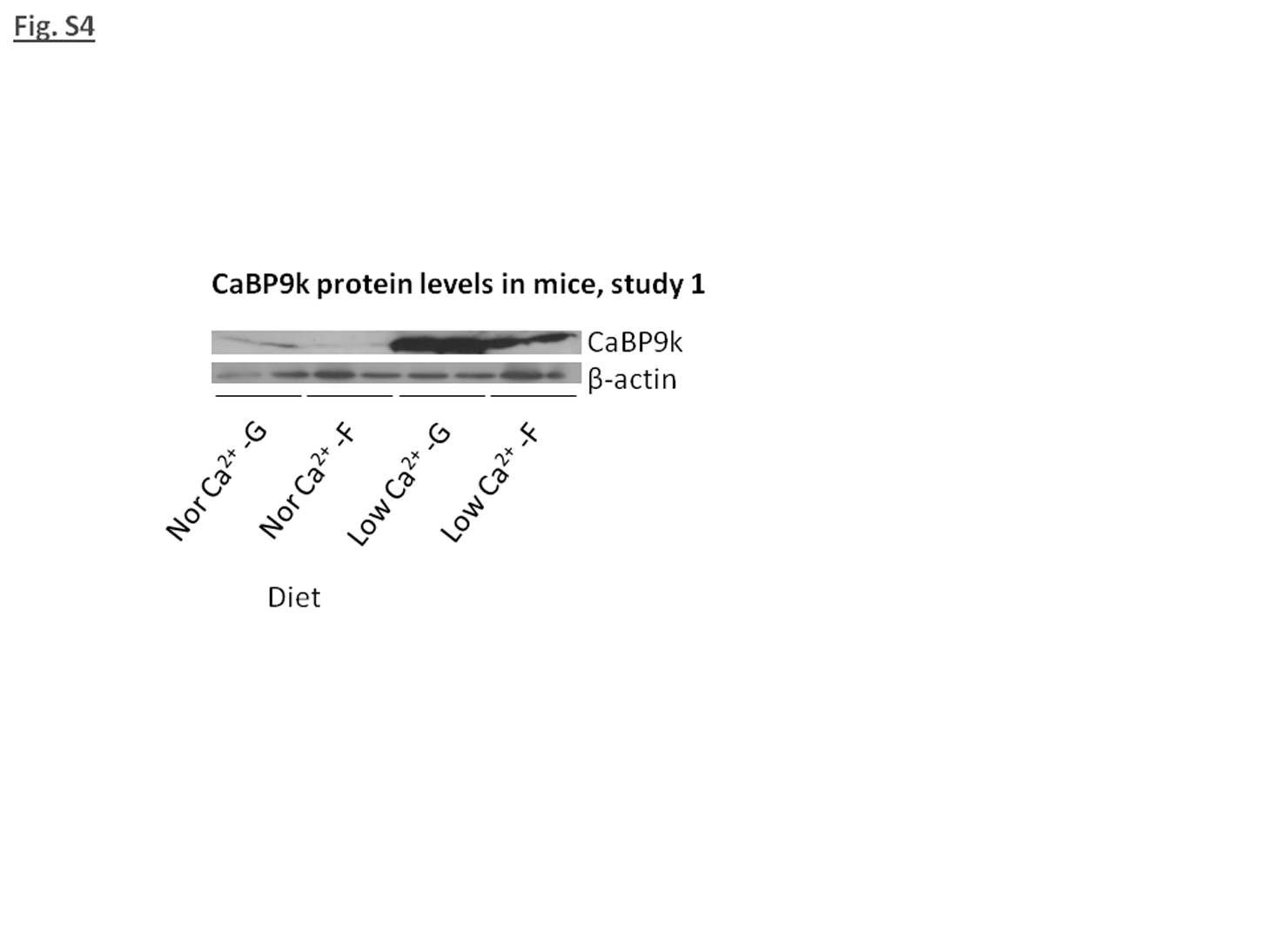

Supplement: Figure S4 — CaBP9k protein abundance. The protein abundance of CaBP9k was determined in the small intestine of mice fed diets containing either 43% glucose or fructose, in combination with either normal or low Ca2+. β-actin was used as a reference. Nor = normal; G = glucose; F = fructose. (TIF) [file pone.0093611.s004.tif]

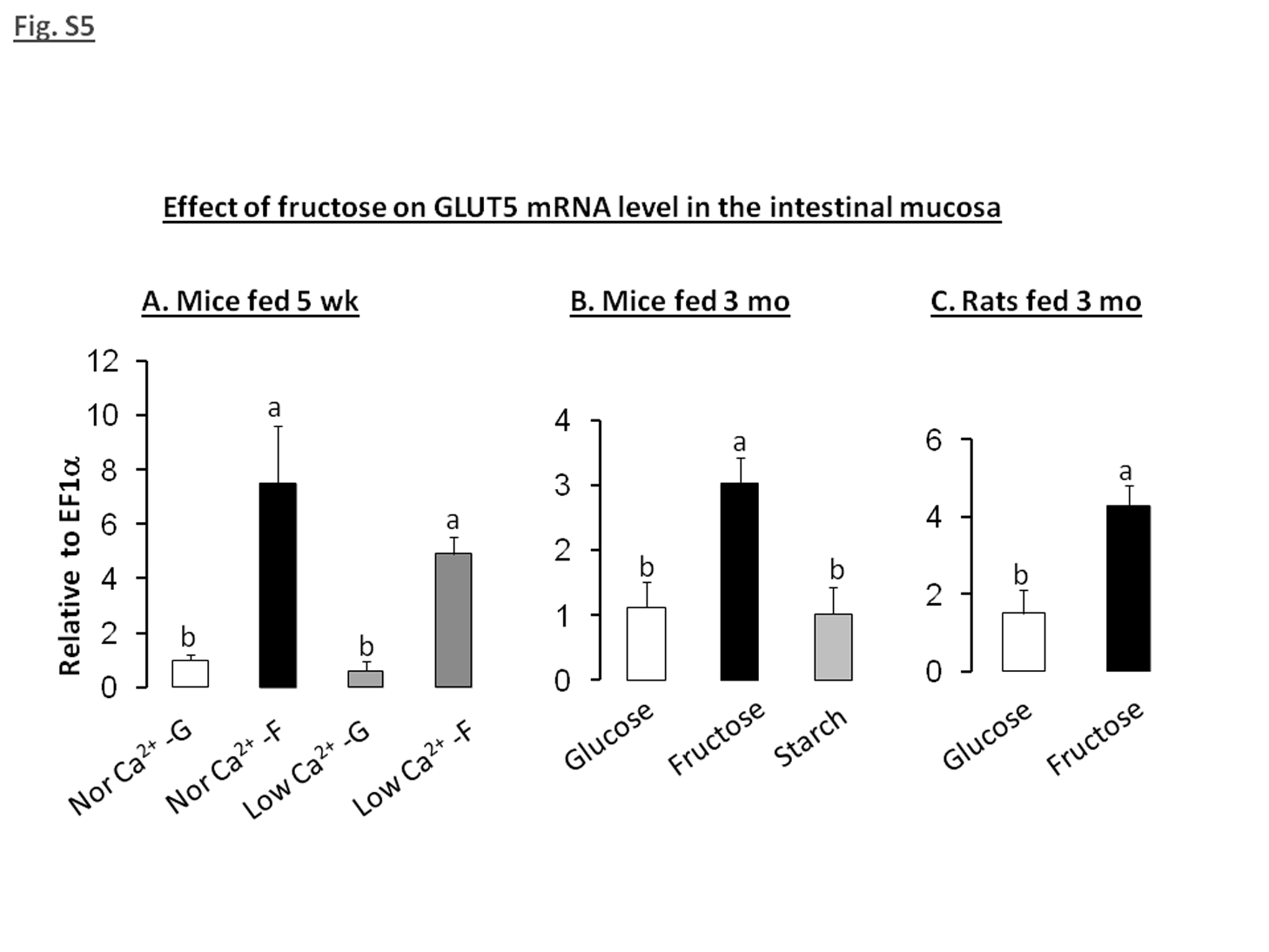

Supplement: Figure S5 — mRNA levels of GLUT5. Expression level of GLUT5 in the intestine of A) mice fed diets containing either 43% glucose or fructose, in combination with either normal or low Ca2+ for 5 wk; B) mice fed diets containing normal Ca2+ in combination with 63% glucose, fructose or starch for 3 mo; and C) rats fed diets containing either 43% glucose or fructose for 3 mo. All expression data were analyzed by real-time PCR using EF1α as a reference and normalized relative to levels seen in rat fed glucose diet. Nor = normal; G = glucose; F = fructose. Data are means ± SEM (n = 6 per group). Differences (P<0.05) among means are indicated by differences in superscript letters, as analyzed by 1-way ANOVA LSD. Thus, for each study, bars with superscript “a” are > bars with “b”. (TIF) [file pone.0093611.s005.tif]
