# Supplementary material for: Chronic High Fructose Intake Reduces Serum 1,25 (OH)2D3 Levels in Calcium-Sufficient Rodents
Source: PLoS One. 2014 Apr 9;9(4):e93611. doi: 10.1371/journal.pone.0093611 (PMC3981704; doi:10.1371/journal.pone.0093611)
Supplement: Table S1 — Primer sequences. (DOC) [file pone.0093611.s006.doc]

**Table S1**: Primer sequences

| Gene name | Forward primer | Reverse primer | Annealing  temperature | amplicon length |
| --- | --- | --- | --- | --- |
| **Rat primers** | | | |  |
| Rat CaBP9k | gacctcacctgttcctgtctg | gctccttcttctggcttcatt | 56 | 316 |
| Rat CaBP28k | gaaggaaaggagctgcagaa | ttcatctcaggtgatagctcca | 56 | 83 |
| Rat CYP24A1 | TGGATGAGCTGTGCGATGA | TGCTTTCAAAGGACCACTTGTTC | 55 | 75 |
| Rat CYP27B1 | caactcgggggttaactaacag | aagcatggaaggatcagtgg | 57 | 69 |
| Rat EF1α | CTCCACTTGGTCGTTTTGCTGT | AGACTGGGGTGGCAGGTGTT | 59 | 165 |
| Rat GLUT5 | TGCAGAGCAACGATGGAGAAA | ACAGCAGCGTCAGGGTGAAG | 59 | 220 |
| Rat PMCA1 | tggaatttgcaccaagttga | caccttcttcttccccaaca | 53 | 272 |
| Rat TRPV5 | gctttcctccaagaagatagagg | gggttgtccatatttcttccac | 56 | 94 |
| rat TRPV6 | caagatctcaacagacaacgc | tagatctggtactcccagccctc | 56 | 278 |
| **Mouse primers** | | | |  |
| Mice CaBP9k | aaatatgcagccaaggaagg | cagctccttaaagagattgtcca | 56 | 126 |
| Mice CaBP28k | gacggaagtggttacctgga | atttccggtgatagctccaa | 55 | 98 |
| Mice CYP24A1 | cctgggacaccattttcaa | caaaggaaatccgcacca | 54 | 83 |
| Mice CYP27B1 | agtggggaatgtgacagagc | ggagagcgtattggataccg | 55 | 61 |
| Mice EF1α | acacgtagattccggcaagt | aggagccctttcccatctc | 55 | 119 |
| Mice GLUT5 | TGCAGAGCAACGATGGAGAAA | ACAGCAGCGTCAGGGTGAAG | 59 | 220 |
| Mice PMCA1 | TGACGATGAACAGGATGACG | CCAAGAGAAACCCCAACAAG | 54 | 225 |
| Mice TRPV5 | cctggatgctgctataatgct | tgtgcagtgcagtctgaccta | 55 | 99 |
| Mice TRPV6 | gctgatggctgtggtaattct | gggatcctctgtctggaaaa | 56 | 67 |
